# Sensors, Signal and Image Processing in Biomedicine and Assisted Living

**DOI:** 10.3390/s20185071

**Published:** 2020-09-07

**Authors:** Dimitris K. Iakovidis

**Affiliations:** Department of Computer Science and Biomedical Informatics, University of Thessaly, 35131 Lamia, Greece; diakovidis@uth.gr

Sensor technologies are crucial in biomedicine, as the biomedical systems and devices used for screening and diagnosis rely on their efficiency and effectiveness. In this context digital signal and image processing methods play an important role in feature or quality enhancement, and compression of the acquired, transmitted, or received biomedical signals and images. Today, smart sensor systems, incorporating such methods, are entering our life through smartphones and other wearable devices to monitor our health status and help us maintain a healthy lifestyle. The impact of such technologies can be even more significant for the elderly, or for people with disabilities, such as the visually impaired.

This special issue gathers a broad range of novel contributions on sensors, systems, and signal/image processing methods for biomedicine and assisted living. These include methods for heart, sleep and vital sign measurement [1,2,3,4,5]; human motion-related signal analysis in the context of rehabilitation and tremor assessment [6,7,8]; assistive systems for color deficient and visually challenged individuals, as well as for wheelchair control by people with motor disabilities [9,10,11,12]; and, image and video-based diagnostic systems [13,14,15,16].

## 1. Heart, Sleep, and Vital Sign Measurement

Contactless measurement of biomedical signals, *i.e.*, measurement performed from a distance from the subjects, are more hygienic, and usually, they can be performed faster on larger populations. A methodology for distant heart rate measurement is presented in [1]. It aims to continuous monitoring of the user’s heart rate during typical human-computer interaction (HCI) scenarios, *e.g.*, during reading texts and playing games. Monitoring is based on the principles of video plethysmography (VPG), a non-invasive, low-cost technique used to detect volumetric changes in the peripheral blood circulation, using a high frame rate RGB camera. Novel contributions of that study include experimental assessment of the impact of human activities during various HCI scenarios, and a novel image representation enabling more accurate and faster measurements.

Another novel contactless heart rate measurement approach is presented in [2], where heart rate is detected with a continuous-wave Doppler radar and an artificial neural network (ANN). Its experimental evaluation on healthy volunteers indicates that this approach is viable for the fast detection of individual heartbeats without heavy signal preprocessing.

To overcome the drawbacks of infrared thermography, which is characterized from low sensitivity for fever-based screening, a novel measurement system combining RGB with thermal image sensors is presented and evaluated in [3]. This system measures multiple vital signs, including body temperature, heart rate and respiration rate, contactlessly. Another contribution of that study is the analysis of the acquired signals using a machine learning methodology to discriminate patients with seasonal influenza from healthy individuals.

A novel methodology for adaptive sampling of electrocardiographic (ECG) signals is proposed in [4]. In that study generalized perceptual features, extracted from the statistics of experts’ scanpaths obtained by gaze tracking, are used to control the ECG sampling process. The main advantage of the proposed method is that the temporal distribution of the distortions, caused by local data loss of the ECG trace, is based on medical features of the signals. Its experimental evaluation indicates a compression efficiency suitable for clinical applications.

Sleep-related metrics from different sleep smart monitoring devices, including a smartwatch, a mattress sensor pad, and a smart ring, are comparatively assessed in [5]. The metrics address sleep staging and total sleep duration, and they are derived via proprietary algorithms that utilize various physiological recordings. The results of that study indicate moderate correlations between the different devices, which indicates that there are still open issues with respect to standardization of such metrics across different devices.

## 2. Human Motion-Related Signal Analysis

Human motion analysis can be useful for the diagnosis and management of various medical conditions, such as the Parkinson’s disease (PD). In [6], a machine learning -based method for automatic assessment of human motor behavior from video, is proposed. Novel contributions of that work include the development of a robust hierarchical multiple-target pose tracking method in uncontrolled environments in the presence of multiple human actors; the introduction of an explicit body movement representation, called pose evolution, that can be used to complement appearance and motion cues for action recognition; and, a target-specific action classification architecture applied on video recordings of patients with PD. The experimental evaluation of the proposed method indicates that it can provide accurate target-specific classification of activities in the presence of other human actors, robust to changing environments.

In a relevant context, [7] proposes a novel device, called the Rehapiano, for the fast and quantitative assessment of action tremor, such as PD tremor. The device uses strain gauges to measure force exerted by individual fingers, and it is applied and assessed for the measurement and monitoring of the development of upper limb tremor.

Signals related to human motor activities can also be obtained by electroencephalography (EEG). In fact, EEG can provide information not only about motor activities that actually happen, but also about motor activities that are imagined by a human subject. The work presented in [8] presents a methodology for improved classification of such motor imagery signals. It is based on a combination of a blind source separation to obtain estimated independent components, a 2D representation of these component signals using the continuous wavelet transform, and a Classification Stage Using A Convolutional Neural Network (CNN).

## 3. Assistive Systems

Assistive systems can have a significant impact on the quality of life of individuals with disabilities or deficiencies. A novel application for improving color discrimination for individuals with color vision deficiency is presented in [9]. In that study, the proposed approach is to automatically recolor the images by a color warping technique, so that the different colors of an image become perceivable by individuals with color vision deficiency.

To assist outdoor navigation of visually challenged individuals, a novel wearable visual perception system (VPS) is presented in [10]. By wearing that system, which is equipped with a stereoscopic RGB camera that can assess depth, the users receive information enabling them to avoid obstacles and safely navigate in outdoor environments. The proposed system goes beyond the state-of-the-art by following a novel uncertainty-aware approach to obstacle detection, incorporating salient regions generated using a Generative Adversarial Network (GAN) trained to estimate saliency maps based on human eye-fixations. The estimated eye-fixation maps, expressing the human perception of saliency in the scene, adds to the intuition of the obstacle detection methodology.

Video coding and transmission methods can be useful in the context of such assistive systems, *e.g.*, for transmitting the images acquired by the system as a video stream to remote users that could provide navigational instructions to the visually challenged. These methods can be inspired from other application domains, *e.g.*, robotics or vehicular technologies. In this light, a low-complexity H.265/HEVC encoder for ad-hoc vehicular networks, is presented in [11].

In the landscape of assistive systems, challenging, still unresolved issues are associated with wheelchair control. In this context, [12] proposes an intelligent, low-cost eye-tracking system for motorized wheelchair control, *e.g.*, for cases with complete paralysis of the four limbs. The input of that system is images of the user’s eye that are processed to estimate the gaze direction and move the wheelchair accordingly to different directions.

## 4. Image and Video-Based Diagnostic Systems

Medical imaging and medical image interpretation, constitute essential tools for healthcare, contributing to patient safety, usually in a cost-efficient way. Among the recently upcoming medical imaging techniques, hyperspectral imaging is among the most promising for the discrimination of malignant tissues. In this context, [13] investigates the most relevant spectral bands for identification of brain cancer from in vivo brain images, by applying heuristic optimization algorithms. An important outcome of that study is the identification of specific spectral ranges for brain cancer detection.

A CNN-based approach is proposed for hyperspectral analysis of histopathological images, aiming to the detection of glioblastoma tumor cells [14]. The main goal of that work is to differentiate between high-grade gliomas (glioblastoma) and non-tumor tissue. Its results indicate a slight advantage in the use of hyperspectral, instead of RGB images.

In the context of endoscopy, narrow band imaging (NBI) constitutes a promising technique for the discrimination of different kinds of lesions as it can provide intraoperative real-time visualization of the vascular changes in the mucosa. A relevant work [15], presents an automatic approach for classification of laryngeal lesions, based on vascular patterns in Contact Endoscopy NBI images. The proposed approach demonstrated its capacity to act as an aid when there are disagreements among otolaryngologists, or when they all misclassify the patients.

In [16], the use of RGB endoscopic video sequences of the gastrointestinal tract is investigated to improve the detection of early malignant lesions appearing in patients with Barrett’s esophagus. Such lesions are very difficult to distinguish, and they are often missed due to subtle visual features. Unlike previous methodologies that use still endoscopic images for lesion detection, the methodology presented in that study focuses on temporal feature extraction from endoscopic video for enhanced robustness of tissue classification and lesion detection. This is achieved by a two-stage ANN architecture performing feature extraction and classification, based on CNNs and recurrent neural networks. The results validate that the proposed approach improves temporal stability and accuracy of tissue classification.
